# Prevalence and Specificity of RBC Alloantibodies in Indian Patients Attending a Tertiary Care Hospital

**DOI:** 10.1155/2014/749218

**Published:** 2014-10-16

**Authors:** Shamsuz Zaman, Rahul Chaurasia, Kabita Chatterjee, Rakesh Mohan Thapliyal

**Affiliations:** Department of Transfusion Medicine, All India Institute of Medical Sciences, New Delhi 110029, India

## Abstract

*Background.* Red blood cell (RBC) alloimmunization results from genetic disparity of RBC antigens between donor and recipients. Data about alloimmunization rate in general patient population is scarce especially from resource limited countries. We undertook this study to determine prevalence and specificity of RBC alloantibodies in patients admitted in various clinical specialties at a tertiary care hospital in North India. *Methods.* Antibody screening was carried out in 11,235 patients on automated QWALYS 3 platform (Diagast, Loos, France). Antibody identification was carried out with an 11-cell identification panel (ID-Diapanel, Diamed GmbH, Switzerland). *Results.* The overall incidence of RBC alloimmunization in transfused patients was 1.4% (157/11235), with anti-E being the most common specificity (36.3%), followed by anti-D (16%), anti-c (6.4%), anti-c + E (6.4%), anti-C + D (5.1%), and anti-K (4.5%). The highest incidence of alloimmunization was observed in hematology/oncology patients (1.9%), whereas in other specialties the range was 0.7–1%. *Conclusion.* As alloimmunization complicates the transfusion outcomes, authors recommend pretransfusion antibody screening and issue of Rh and Kell matched blood to patients who warrant high transfusion requirements in future.

## 1. Introduction

Red blood cell (RBC) transfusion is a lifesaving therapy for complications of anemia and treatment of the symptoms and signs of hypoxia. However, the risk of RBC alloimmunization is always a concern for patients receiving RBC transfusions [1]. Alloimmunization occurs because of red cells antigenic differences between donor and recipient or between mother and fetus. As no two humans, except identical twins, have the same genetic makeup, blood transfusion exposes the patient to numerous “foreign” antigens. These foreign antigens are potential immunogens which can lead to development of antibodies in the recipient within days, weeks, or months after a transfusion [2].

Alloantibodies may cause hemolytic disease of new born (HDN), hemolytic transfusion reaction (HTR, acute, or delayed), or decrease in the survival of transfused RBCs. Presence of alloantibodies in patients leads to difficulty in finding compatible RBC units and, thus, delay in issuing compatible blood [3]. The prevalence of clinically significant alloantibodies has been reported from less than 0.3% to up to 60% of samples depending on the study populations and the test method sensitivity [4, 5]. Not uncommonly, autoantibodies can also be found along with alloantibodies which have been reported to be as high as 28% [6]. The concomitant presence of auto- and alloantibodies may further complicate serological workup and add to difficulty in obtaining a suitable crossmatch-compatible blood and may result in further decrement in posttransfusion survival of RBCs [7, 8]. Theoretically, risk of alloimmunization can be significantly decreased by typing the donors' and patients' clinically significant antigens. This extended matching would be an ultimate solution, although the associated costs and logistics will raise serious concerns especially in resource limited countries [3]. Moreover, due to different distribution of blood groups in patient and general population, managing inventory in the face of extended-crossmatching will further pose serious challenges [9].

Previously performed studies have largely concentrated on multiply transfused patient populations or antenatal women [10–14]. Data about relative frequency of RBC alloantibodies in the general patient population receiving occasional RBC transfusions has not been studied extensively. In the current study, we analyzed the prevalence and specificity of RBC alloantibodies in patient population from various clinical specialties by employing automated QWALYS 3 system (Diagast, Loos, France) for antibody screening. Antibody screen-positive samples were further analyzed for their antibody specificity.

## 2. Material and Methods

Data of antibody screening between years 2012 and 2013 were retrieved from case records at Department of Transfusion Medicine, All India Institute of Medical Sciences, New Delhi, and assessed for the presence of alloantibodies. During the study period all patients for whom routine transfusion requests were received or any incompatibility was reported were included in the study. All cases underwent antibody screening and if found positive were subjected to antibody characterization/identification. All antenatal women and patients with only autoantibodies were excluded from the study. All cardiac surgery, neurosurgery, and trauma patients were also excluded as these specialties are not catered by our transfusion facility.

### 2.1. Serological Workup

Blood grouping and antibody screening were performed on QWALYS 3 (fully automated system, Diagast, Loos, France) based on Erythrocyte Magnetization Technology. This system uses ABD-Lys and Hemascreen for blood grouping and antibody screening, respectively. The detailed principle and methodology of the system are excellently reviewed by Schoenfeld et al. [15]. Briefly, the system utilizes magnetic hemagglutination and avoids steps of centrifugation and washing. All serum samples positive on automated antibody screen were referred to immunohematology laboratory where antibody identification was performed manually using commercial 11-red cell panel (ID-DiaPanel, BioRad, Switzerland). An autocontrol using the patient's cell and serum was tested in parallel with each screen to exclude presence of autoantibodies.

### 2.2. Blood Transfusion Protocol

Patients with a negative antibody screen received a transfusion of ABO and Rh(D)-compatible RBCs by an immediate spin crossmatch technique. For alloimmunized patients, antigen-negative, crossmatch-compatible RBCs were transfused. The treating clinicians were informed regarding the presence and nature of alloantibody.

### 2.3. Statistical Analysis

The analysis and data management were performed using SPSS software version 16 (SPSS, Inc., Chicago, IL, USA).

## 3. Results

In total, 11235 patients (6573 males and 4662 females, mean age 32.37 years, and range 1–83 years) from various clinical specialties who received packed RBCs were included in the study. Demographic details, ABO and Rh distribution are shown in Table 1. The antibody screen was positive in 215 patients. On further characterization 157 (73%) patients were found to have alloantibodies and 58 (27%) patients with only autoantibodies were excluded from study. Concomitant presence of autoantibodies was found in 9 patients (0.08%). The overall prevalence of RBC alloimmunization was 1.4%. Females had a higher alloimmunization rate of 2.1% versus 0.9% in males; difference was clinically significant (*P* < 0.05). Distribution of patients according to clinical specialties is given in Table 2. The highest number of alloimmunized patients belonged to hematology/oncology group (*n* = 118) with prevalence rate of 1.9%, whereas in other specialties the alloimmunization rate was between 0.7 and 1.0%. A total of 13 different alloantibodies either singly or in combination were identified in 157 patients. Antibodies to the Rh blood group system were the most frequent being present in 120 (76.4%) patients. 19 patients (12.1%) showed presence of multiple alloantibodies. The prevalence of autoantibodies along with alloantibodies was found to be 5.7% (*n* = 9) of total alloimmunized patients. In 12 (7.6%) instances, specificity of antibodies could not be determined. The specificities of alloantibodies identified are shown in Table 3.

## 4. Discussion

RBC alloimmunization results from the antigenic disparity of red cells between donor and recipient or between mother and fetus. Current standard pretransfusion testing protocols require detection and identification of clinically significant antibodies reacting in antihuman globulin (AHG) phase after incubation at 37°C.

In present study, the overall alloimmunization rate was 1.4% which was low when compared with a study done by Thakral et al. who reported prevalence of 3.4% [16]. This difference could be due to varied study populations. In a similar study in Tehran, prevalence of alloimmunization reported was 0.97% which was comparable to our study [17]. Female patients had higher rate of alloimmunization than male in our study (2.1 versus 0.9%, *P* < 0.05). A systematic review by Verduin et al. also showed that women have slightly higher rate of alloimmunization than men although they categorically state that, based solely on sex difference, results do not justify recommending additional matching for women [18]. The high prevalence of alloimmunization in hematology/oncology (*n* = 118, 1.9%) could be due to high incidences of RBC antigenic exposures in this group. In other specialties alloimmunization rate ranged from 0.7 to 1.0%. In a similar study, Schonewille also reported that occasionally transfused patients have alloimmunization rate ranging between 1 and 3% [19]. The most prevalent antibodies in our study were against E (36.3%), D (16.0%), and c (6.4%) and c + E (6.4%), C + D (5.1%), and K (4.5%) antigens. Al-Joudi et al. also reported anti-E as the most common antibody [20]. Study by Thakral et al. also showed 22.2% prevalence of anti-E; however, the most common alloantibody detected by them was anti-c (38.8%) [16]. The differences in antibody specificity could be attributed to the difference in the study population at both centers. In our study of 34 patients with anti-D (either singly or in combination) majority (*n* = 22) were multiparous females who might have formed anti-D due to previous pregnancies or transfusions. The rest of the 12 patients were transfusion dependent due to underlying medical/oncologic conditions and might have received Rh(D)-incompatible transfusions leading to the formation of anti-D in these patients. The underlying clinical conditions of these 12 multiply transfused patients were thalassemia (*n*-6), aplastic anemia (*n*-3), carcinoma (*n*-2), and AIHA (*n*-1). A study by Schonewille et al. evaluated alloimmunization in myeloproliferative and lymphoproliferative diseases and reported 4 (7.8%) patients who formed anti-D antibody [1]. Sadeghian et al. studied the development of alloimmunization among Iranian transfusion-dependent thalassemia patients and found that 8 out of 9 alloimmunized patients formed anti-D in the course of the disease with marked preponderance in female patients [21].

Most of the studies done outside India report incidence of anti-K as high as 23% [17, 22, 23]. Low prevalence of anti-K in our study (4.5%) could be due to low frequency of Kell antigen in Indian population (1.97%) as compared to frequency of 8.8% in Caucasian population [24]. Nineteen (12.1%) alloimmunized patients showed presence of multiple antibodies. Al-Joudi et al. also reported multiple antibodies in 23.1% of patients [20]. Since pretransfusion antibody screening in patients' samples is not a routine practice in India, these patients might have received antigen-mismatched blood leading to formation of multiple alloantibodies. Unfortunately, the records of previous transfusions received elsewhere were not available to us. Nine patients (5.7%) had coexisting autoantibodies along with alloantibodies. Ahrens et al. had reported increased risk of autoantibody formation in face of concomitant alloimmunization [6]. We were not able to determine the specificity of antibody(ies) in 12 (7.6%) patients. This may be due to lack of indigenous red cell panels [25]. Salamat et al. also emphasized that red cell panels sourced from local population would be better for detection of antibodies as cell panels from nonindigenous populations may miss certain antibodies against antigens in local population [26]. The frequency of RBC alloantibodies varies considerably depending upon numerous factors, for example, demographics, number of transfusions, pregnancy, genetic constitution, immune competence, disease factors, time and frequency of screening, and sensitivity of the methodology [8, 27]. Although the patients from other specialties were not exempt from the risk of alloantibody formation, we found the highest proportion of alloimmunized patients in hematology/oncology group. The issue of routine antibody screening of all patients requiring transfusion, that too, in resource limited countries is highly debatable [28]. Thus, for prevention of alloimmunization, authors recommend the transfusion of Rh and Kell antigen-matched blood to those patients whose natural history of disease dictates high transfusion requirements in future.

## Figures and Tables

**Table 1:** Demographic profile of study population.

	Total patients *n* = 11235	Alloimmunized patients *n* = 157
Gender		
Male	6573	57
Female	4662	100

Age group (years)		
<10	2427	9
11–20	2168	37
21–30	1831	32
31–40	1393	30
41–50	1180	21
51–60	1292	19
>60	944	9

ABO group distribution		
O	3449	56
A	2764	25
B	3910	52
AB	1112	24

Rh group distribution		
Rh D positive	10392	115
Rh D negative	843	42

**Table 2:** Distribution of patients according to clinical specialties.

Specialty	Total number of patients	Mean age	Alloimmunized patients	Auto- + alloantibody	Sex M/F
Hematology/oncology	6282	41.6 ± 17.6	118 (1.9%)	7	46/72
Gynecology	964	39.8 ± 18.8	8 (0.8%)	0	0/8
Orthopedics	1575	43.75 ± 14.1	11 (0.7%)	0	5/6
Nephrology/urology	923	33.6 ± 11.7	8 (0.9%)	0	3/5
Gastroenterology/gastrosurgery	908	35.5 ± 12.0	6 (0.7%)	0	1/5
Others	583	30.1 ± 11.2	6 (1%)	2	1/5

Total	11235		157 (1.4%)	9 (0.08%)	

**Table 3:** Specificities of alloantibodies.

Antibody(ies)	Number of patients	Percentage
Alloantibodies		
Anti-c	10	6.4
Anti-C	3	1.9
Anti-c and anti-E	10	6.4
Anti-C + D	8	5.1
Anti-C + D + E	1	0.6
Anti-D	25	16.0
Anti-e	1	0.6
Anti-E	57	36.3
Anti-Fy^a^	1	0.6
Anti-Jk^a^	3	1.9
Anti-K	7	4.5
Anti-Kp^a^	1	0.6
Anti-Le^a^	4	2.5
Anti-Lu^a^	1	0.6
Anti-M	4	2.5

Auto- + alloantibodies		
Auto- + alloanti-c	2	1.3
Auto- + alloanti-E	3	1.9
Auto- + alloanti-K	3	1.9
Auto- + alloanti-S	1	0.6
**Not determined**	12	7.6

Total	157	≈100%
